# Effects of zooplankton abundance on the spawning phenology of winter-spawning Downs herring (*Clupea harengus*)

**DOI:** 10.1371/journal.pone.0310388

**Published:** 2025-02-05

**Authors:** Paul Marchal, Carolina Giraldo, David Johns, Sébastien Lefebvre, Christophe Loots, Lola Toomey

**Affiliations:** 1 Channel and North Sea Fisheries Research Unit, Institut Français pour la Recherche et l’Exploitation de la Mer, Boulogne s/mer, France; 2 The Laboratory, Marine Biological Association, Plymouth, Devon, United Kingdom; 3 Laboratoire d’Océanologie et Géosciences, Université de Lille, Centre National pour la Recherche Scientifique, Université du Littoral Côte d’Opale, Institut pour la Recherche et le Développement, Lille, France; 4 Coispa Technology and Research, Bari, Italy; Havforskningsinstituttet, NORWAY

## Abstract

We have investigated phenological shifts in autumn- and winter-spawning Atlantic herring (*Clupea harengus*) in the Eastern English Channel and the Southern North Sea (Downs component), in relation to temperature and the availability of potential zooplanktonic prey (*Calanus finmarchicus*, *Calanus helgolandicus*, *Temora longicornis*). A two-tiered approach building on the monthly distribution of commercial herring landings was developed, which consisted of, (1) calculating the timing and duration of spawning season based on estimated deviations from basic harmonic signals and, (2) analysing their inter-annual variations in relation to biotic (zooplankton abundance) and abiotic (temperature) environmental variables through time series analyses. The start, midpoint and ending of herring spawning season were increasingly delayed over the period 1999–2021, a process which was correlated with the abundance of *Calanus finmarchicus*. The resulting duration of spawning season slightly decreased. Direct effects of sea temperatures on any phenological metrics could not be clearly evidenced. Different ecological processes were likely involved in the start and ending of spawning season. Additional covariates (including size/age composition, the biotic and abiotic factors other than those examined in our study) could contribute to a better explanation of the phenological drift in Downs herring spawning.

## Introduction

Phenological changes are recognized as a major symptom of global warming affecting terrestrial and marine organisms [1], with knock-on effects on the structure of ecosystems, and the economic viability of human activities exploiting them [2, 3]. Phenological responses are largely driven by temperature in many mid-latitude taxa [4, 5]. In the seas, spawn-stage fish are particularly at risk since their thermotolerance is lower than other life-stages [6, 7]. In this way, increased water temperatures have been shown to affect fish spawning phenology, either directly by acting on the sexual maturation process [8–11], or indirectly by impacting their prey availability [12–15]. Most mid-latitude marine fish spawn in spring, with accumulating evidence that spawning has been advanced as a direct or indirect effect of increased temperatures [8, 11, 16–19]. The duration of spawning seasons has also been impacted by global warming, with an extension sometimes suggested for spring-spawning species [11, 20].

Some fish populations spawn in fall or winter, however, these represent relatively rare cases compared to spring-spawners. Despite generally less favourable environmental conditions, particularly in terms of food availability, the cold seasons could offer a refuge to spawning fish and their offspring, where the competition for prey and predation pressure could perhaps be less intense than in spring [21, 22]. In the case of autumn- or winter-spawning anadromous fish, increased temperatures affected the temporal window of spawning in different ways depending on the species, population or age class being considered [23–25]. The spawning phenology and duration of autumn- or winter-spawners has rarely been investigated in the case of marine fish (but see [26, 27]).

A substantial change in the timing and duration of spawning season could have adverse effects on reproductive success, offspring survival and eventually impair subsequent recruitments. This is particularly true in the case of planktivorous fish larvae which could shift out of phase with plankton abundance peaks, leading to starvation and potential mortality [2, 28, 29].

We investigated phenological shifts in the winter-spawning season of the Downs component of Atlantic herring (*Clupea harengus*) (hereby referred to as Downs herring), which is the most southerly distributed spawning component of the North Sea herring stock (International Council for the Exploration of the Sea, ICES, Subarea 4 and Divisions 3a and 7d). The phenology of winter-spawning Downs herring has not been investigated as extensively as in other spring-spawning fish, and our study addresses this particular knowledge gap. We investigated here the phenological response of Downs herring, characterized by changes in the start, midpoint, ending and duration of spawning migrations, to both abiotic (i.e. temperature) and biotic (i.e. zooplankton prey abundance) environmental factors using a novel two-tiered approach.

A variety of studies have investigated seasonal aspects of plankton or fish population dynamics, including phenological changes. In most studies we are aware of, phenological shifts have been evidenced using different empirical metrics (e.g., indicator of central tendency, timing of the initial increase in abundance, timing at which abundance reaches some cumulative percentile of total annual abundance) applied directly to the data series without making explicit the background seasonal signals [3, 11, 17, 30, 31]. However, unplanned changes in sampling frequency over time and space could bias the derivation of such empirical metrics, particularly when sampling is opportunistic or when information is missing, which is often the case in fisheries and ecosystem data collected *in situ*. An alternative approach to explicitly investigate periodic dynamics, applied in various biological and epidemiological research investigations, has been to build in harmonic functions in statistical models, ranging from simple sine waves [32–34], to more complex representations [35–37]. Although fitting harmonic models is not immune to missingness or uneven temporal data distribution, this modelling approach is likely less sensitive to such biases, compared to empirical metrics calculated directly from averaging raw data. While the scope of the aforementioned studies was not to focus on phenology shifts, the approach we developed in this paper builds on similar harmonic models to explicitly mimic the strong background periodic signal inherent to pelagic dynamics, and to refine it by including additional parameters to estimate annual changes in both phenology and abundance, in a Generalized Additive Modelling (GAM) framework. In our study, we analytically derived four indicators reflecting the midpoint, start, ending and duration of Downs herring spawning season from the estimated harmonic functions. We modelled to that purpose monthly-distributed coastal landings from the French pelagic fishery available over the period 1999–2021. In a second stage, we modelled the previously calculated phenological indicators in relation to zooplanktonic prey abundances and Sea Surface Temperatures (SST) anomalies. A time series analysis building in transfer functions was performed so to identify instantaneous, but also lagged effects of environmental changes on herring spawning phenology.

## Materials and methods

### Case study

The North Sea herring stock (ICES Subarea 4 and divisions 3a and 7d) is structured mainly in four large components spawning in autumn and winter: Orkney/Shetland (spawning off eastern Scottish Islands), Buchan (spawning off eastern Scotland), Dogger (spawning in the central North Sea) and Downs (spawning in the Eastern English Channel and the Southern North Sea, EEC-SNS). After hatching, Downs herring larvae are transported to the Eastern North Sea, where they grow for about two years. Adult herring then move onto their feeding grounds located in the northern North Sea, where the four populations mix in spring and summer. The spawning season of the Downs herring population, which this study will focus on, starts in November and finishes in December or January [38, 39]. Spawning fidelity is assumed [40].

Since the collapse of the North Sea herring fishery in the end of the 1970s, the North Sea stock has gradually recovered, and ICES considers it has been exploited sustainably since the mid-1990s [41]. After almost disappearing in the end of the 1970s, the Downs population (ICES Divisions 4c and 7d) has become a strong component of the total North Sea herring stock, contributing to about 25% of its spawning biomass in 2021 [39]. The Downs herring fishery is mainly operated between November and January by Dutch, French, German and English vessels. The French fishery, which is the main herring data provider for this study, is mainly operated by pelagic trawlers, including few large vessels fishing in the whole North Sea and smaller-sized coastal vessels harvesting almost exclusively the Downs component. An annual average of 9,500 t of herring were landed by French pelagic trawlers operating in ICES Divisions 4c and 7d over the period 2018–2022.

### Data

#### Herring landings

Detailed information on the duration of fishing trips, vessel length and pelagic species landings were made available from fishers’ mandatory logbooks over 1999–2021 for the EEC-SNS. Only fishing trips operated by pelagic otter- or pair-trawlers, for which the duration was less than two days were retained (S1 Fig). These filters were applied to circumscribe the geographical coverage of the dataset to the autumn-winter distribution of Downs herring. In order to differentiate between situations where herring was not landed due to a lack of selectivity of the fishing gear or due to low abundance (e.g., off the autumn and winter seasons), we also considered landings from other pelagic species normally caught by pelagic gears: mackerel (*Scomber scombrus*), horse mackerel (*Trachurus trachurus*), and sardine (*Sardina pilchardus*). For instance, pelagic trawlers operating in the EEC-SNS in July target mackerel but catch almost no herring. Most logbooks registered in July would therefore report mackerel but not herring in the species caught. We therefore added a line for herring landings (with a 0 value) wherever a fishing trip reported mackerel, horse mackerel or sardine, but not herring. We then derived daily landings (tonnes/day) for each fishing trip. While herring landings would not be expected to reflect annual biomass levels due to inter-annual variations in fishing pressure, we assumed here that fishers had perfect knowledge of herring schools which they could harvest with limited constraints, so the seasonal patterns in herring landings could reasonably reflect the spawning phenology of Downs herring [42, 43].

Downs herring spawn between November and January in the EEC-SNS. To avoid having spawning season overlapping two consecutive years, we backshifted the landings time series by seven months. For instance, July in calendar year 2000, January in calendar year 2001 and June in calendar year 2001, become respectively months 0, 6 and 11 in annual cycle 2000/2001. Daily landings per fishing trip from 22 annual cycles covering the period 1999/2000-2020/2021 were then available to our study.

#### Environmental biotic and abiotic time series

Herring is an extreme capital breeder, meaning that adults store energy reserves that will be used at a later time for spawning [44]. We focused on the food adult herring may have consumed in the northern North Sea in spring and summer, corresponding to the main feeding window of the species [39, 45, 46], and on the sea temperatures individuals may have encountered in that season, prior to their southerly autumn-winter spawning migration.

Although adult herring diet composition in spring and summer is subject to strong spatial, inter- and intra-annual fluctuations, it is dominated by copepods (e.g., *Calanus* spp., *Temora* spp.) [12, 47, 48]. We extracted zooplankton abundance data from the Continuous Plankton Recorder (CPR) programme [49]. The CPR provides a unique source of plankton information, with high spatial and time resolutions. Sampling is carried out by ships of opportunity on a voluntary basis, using the same protocol since 1958. The towed unit includes a passively advancing silk, which traps planktonic organisms as well as smaller-sized particles. The silks are preserved in formalin, split in sections corresponding to 10 nautical miles of tow. Phytoplankton and zooplankton organisms are then identified and counted with a microscope in the laboratory using consistent methodology [49, 50]. We extracted CPR data between April and September and we also selected in the North Sea the spatial units included in the B2 and C2 CPR Standard Areas (S1 Fig), which best corresponded to Downs herring spring and summer feeding grounds [39, 45, 51]. We focused in this study on the main zooplankton taxa that could provide food to North Sea herring in spring and summer (*Calanus finmarchicus*, *Calanus helgolandicus* and *Temora longicornis*), for which abundance indices were averaged over the B2 and C2 CPR area [50]. It was in particular thought important to distinguish between *Calanus finmarchicus* and *Calanus helgolandicus*, which have been subject to contrasted spatial and temporal dynamics in the past decades [52–54].

Zooplanktonic abundance trends and seasonal patterns are shown (Figs A and B in S1 Text) over the period 1980–2020, which includes the period preceding the availability of herring landings data (1999–2021). This is to put the investigation of phenological variations in Downs herring spawning season in a broader temporal and environmental context.

April to September Sea Surface Temperatures Anomalies in North Sea herring feeding grounds (SSTAs), as well as October to December Sea Surface Temperatures Anomalies in Downs spawning grounds (SSTAw)were extracted, for the period 1980–2021, from the US National Oceanic and Atmospheric Administration (NOAA) Optimum Interpolation SST dataset (https://psl.noaa.gov/data/gridded/data.noaa.oisst.v2.html). In our study, SSTAs was averaged between April and September over the B2 and C2 CPR Standard Areas covering the main North Sea herring feeding grounds, while SSTAw was averaged between October and December over an area covering EEC-SNS spawning grounds exploited by the pelagic fishery in that season (S1 Fig).

#### Correlation within and across environmental variables

We examined the temporal structure inherent in the different environmental variables (SST anomalies, zooplankton abundances), and the common patterns they may share, which could blur the interpretation of their possible effect in driving spawning phenology. This preliminary analysis was carried out by checking temporal autocorrelation within and cross-correlation between the environmental time series over the period 1999–2020. As shown in S1 Text, none of the environmental factors being investigated were subject to significant autocorrelation at any time lag. Cross-correlation were found at lag 0, and not for any other lag, between the abundances of *Calanus finmarchicus* and *Calanus helgolandicus* (positive cross-correlation), and also between the abundance of *Temora longicornis* and SST anomalies in April-September over herring feeding grounds (negative cross-correlation). None of the other pairs of environmental variables were subject to significant cross-correlation at any time lag.

### Models

#### Modelling seasonal and annual fluctuations of winter-spawning herring landings

Herring landings variations in the EEC-SNS were modelled using Generalized Additive Models building in a combination of harmonic regression functions to reflect seasonal patterns, and of non-linear processes to accommodate inter-annual variations and temporal auto-correlation in annual landings. The distribution of herring landings, which consisted of many zeroes and of positive values, was here assumed to follow a Tweedie distribution. The Tweedie distribution is characterized by a mean (*μ)*, and a variance (*ϕ μ*^*p*^), where *ϕ* is the dispersion parameter, and *p* is the Tweedie power parameter. Assuming Tweedie distributions combined with logarithmic link functions, the seasonal signal inherent to herring landings was mimicked by the sum of trigonometric functions, representing Fourier harmonics, with time-dependent coefficients reflecting inter-annual variations in seasonal patterns. A thin plate spline term was added to represent overall inter-annual landing fluctuations and to account for temporal autocorrelation. For each observed fishing trip *i*, the expected value of herring daily landings *E[L*_*i*_*]* could be formulated by Eq (1):

$$E\left[L_{i}\right]=exp\left\{\beta_{0}+\sum_{n\geq 1}\left\{\beta_{c,n,y}\;\cos\left(\frac{\text{n}\pi \text{t}}{6}\right)+\beta_{s,n,y}\;\sin\left(\frac{n\pi t}{6}\right)\right\}+sp(y)\right\}$$ (1)

Where *β*_0_ is a constant base line representing the mean response over *N* annual cycles *y*; *t* is the month within each annual cycle; *β*_*c*,*n*,*y*_ and *β*_*s*,*n*,*y*_ are the (annual cycle-dependent) coefficients associated to the *n*-th harmonic function; and *sp(y)* is a spline term reflecting non-linear annual effects. In the simplest case where *n* = 1, expected landings are symmetric around a unique maximum within each annual cycle. Increasing *n* adds more flexibility, and more realism, in the seasonal distribution of expected landings, but it also results in increased model complexity with additional parameters to be estimated. With *N* = 22 annual cycles, each extra harmonic function requires estimating an additional 44 (*β*_*c*,*n*,*y*_ and *β*_*s*,*n*,*y*_) coefficients (in addition to *β*_*0*_ and the smoothing parameters associated to *sp(y)*). We applied Eq (1) with values of *n* increased from 1 to 4. With 4 harmonic functions (*n* = 4), 44 × 5 = 220 harmonic coefficients are to be estimated. This is the maximal number of harmonic functions which could be considered so the total number of parameters to be estimated does not exceed 264, i.e., the total number of months in the herring landings time series (22 annual cycles times 12 months).

Daily landings also depend on vessel characteristics, i.e., large vessels are expected to catch more herring than small vessels. Vessel length was included as an additional explanatory variable in Eq (1) in preliminary analyses, however, without improving the model’s goodness of fit. This is due to the strong decrease of vessel length over the years (Fig in S2 Text), and the resulting collinearity between the vessel length effect and the year effect that is already built in Eq (1). Therefore, the year effect not only comprises annual variations in herring biomass, but also in vessel size composition. The monthly distribution of vessel length slightly fluctuated without trend between 1999/2000 and 2007/2008, and stabilized thereafter.

We considered here that the seasonal distribution of EEC-SNS herring landings dynamics could reasonably reflect the spawning phenology and duration of Downs herring, an assumption which was supported by knowledge on the pelagic fisheries characteristics and management (see Discussion). In contrast, inter-annual herring landings variations were more difficult to interpret and these were represented in the spline effect term.

Model parameters set in Eq (1) were estimated using the PROC GAMPL procedure of SAS/STAT statistical package, Version 9.4 (SAS Institute Inc., Cary, NC, USA). The models’ goodness of fit was evaluated using both the Akaike and the Bayesian Information Criteria (AIC and BIC, respectively).

#### Calculating the spawning phenology and duration of Downs herring spawning season

A numerical approach building on percentage thresholds of total cumulated landings was applied to derive annual phenological indicators of Downs herring spawning season from the estimated parameters of Eq (1). Within each annual cycle, we defined the start, midpoint and ending of Downs herring spawning season as the time that cumulated expected landings, derived from Eq (1), exceeded respectively 25%, 50% and 75% of the total expected landings cumulated over the whole annual cycle. Within each annual cycle *y*, *θ*_*1*,*y*_ (start of spawning season), *θ*_*y*_ (midpoint of spawning season) and *θ*_*2*,*y*_ (ending of spawning season) were calculated by integrating numerically Eq (1) with respect to *t*. Numerical integrations were performed by applying functions NIntegrate and NDSolve of Mathematica software, Version 13.3 (Wolfram Research, Inc.). Finally, the spawning season duration per annual cycle (*δ*_*y*_) was calculated as the difference between *θ*_*2*,*y*_ and *θ*_*1*,*y*_. Spawning peak timing could also in principle be considered as an additional phenological metric by choosing for each annual cycle the apex of the predicted landing curve. Choosing the apex of the curve would be appropriate if the seasonal patterns were unimodal and/or symmetric, which was, however, not the case here. This was tested in preliminary analyses, which showed that the apex would remain almost constant in periods and then vary erratically from one annual cycle to another in other periods. To evaluate the sensitivity of our results to the percentages of total predicted landings chosen to define the start and the end of the spawning season (25% and 75%, respectively), we repeated our analyses with alternative values (5% and 95% for the start and the end of the spawning season, respectively).

Finally, non-linear trends in the inter-annual changes in the different spawning phenology metrics were visualized by fitting a cubic spline smoother, using the PROC LOESS procedure of SAS/STAT statistical package, Version 9.4 (SAS Institute Inc., Cary, NC, USA).

#### Modelling annual changes in the spawning phenology and duration of Downs herring in relation to environmental fluctuations

We modelled the temporal dynamics of the spawning phenology and duration of Downs herring in relation to environmental parameters via an ARMAX time series analysis, to account for both annual auto-correlation in the different variables being investigated and cross-correlation over several time lags between phenological indicators and environmental parameters [55]. The ARMAX model used to mimic the time variations of variable *Z*_*y*_ (which represents here the annual series of the midpoint, start, ending or duration of spawning season), in relation to the 5 potential covariate variables *X*_*1*,*y*_, …, *X*_*5*,*y*_ (which represent here the two annual series of SST anomalies and of the three selected zooplankton indices) could be formulated as in Eq (2):

$$Z_{y}=v+\eta_{y}+\sum_{i=1}^{p}\varphi_{i}Z_{y-i}+\sum_{i=1}^{q}\gamma_{i}\eta_{y-i}+\sum_{j=1}^{5}\sum_{i=0}^{r_{j}}\omega_{i,j}X_{j,y}$$ (2)

Eq (2) is composed of five additive elements: (1) an intercept (*v*); (2) a normally-distributed random noise *η*_*y*_; (3) a *p*-order auto-regressive (AR) term parameterized by *p* linear coefficients *φ*_*i*_ applied to *Z*_*y-i*_ (*i* = 1, 2, …, *p*); (4) a *q*-order moving average (MA) term parameterized by *q* linear coefficients *γ*_*i*_ applied to *η*_*y-i*_ (*i* = 1, 2, …, *q*) and finally; (5) an input transfer function relating *Z*_*y*_ to its 5 potential covariates considering *r*_*j*_ time lags and *r*_*j*_ linear coefficients *ω*_*j*_ applied to each covariate *X*_*j*,*y*_ (representing the “X” in “ARMAX”). Only lag values of *p*, *q* and *r* lower than 2 were considered to allow meaningful ecological interpretation of the results. Note that more complex time series models could have been considered to allow differencing the initial time series in case obvious (e.g., linear) time trends were detected, or using a rational transfer function (i.e., by including a denominator in the fifth component of Eq (2). However, Eq (2) proved sufficient to model the phenological processes under investigation, and that was used in subsequent analyses.

The calibration of each ARMAX should be carried out in five stages: (1) parameterize an ARMA model to each input (environmental) time series (*X*_*j*,*y*_) based on auto-correlation and partial auto-correlation functions; (2) for each input series, use the same ARMA filter defined at stage (1) to pre-whiten both *X*_*j*,*y*_ and *Z*_*y*_; (3) calculate the cross-correlation function between the two series; (4) parameterize the ARMAX model for the explained (phenological) time series (*Z*_*y*_) based on auto-correlation and partial auto-correlation functions and also building on the cross-correlation between (*Z*_*y*_) and the different input time series (*X*_*j*,*y*_) and; (5) select the final ARMAX model based on Akaike (AIC) criterion. Because none of the environmental time series were autocorrelated, stages (1) and (2) became unnecessary. When several significant environmental variables were selected in step (5), we investigated possible collinear effects by removing one covariate at the time and assess the model response in terms of parameterization and outputs. The calibration of the ARMAX models was performed using the PROC ARIMA procedure from the SAS/ETS time series analyses package, Version 9.4 (SAS Institute Inc., Cary, NC, USA).

## Results

### Seasonal and annual fluctuations of winter-spawning herring landings

The statistical diagnostics output from the GAM applied to herring landings are reported in Table 1. All parameters were statistically significant (p < 0.01). The model which achieved the lowest AIC and BIC was the model building in three Fourier harmonic functions. A model with four harmonic functions was explored, but it did not converge after 200 iterations and results are not shown here.

**Table 1:** Number of observations and outputs summary from the Generalized Additive Modelling of herring landings, assuming a Tweedie distribution, and building on Eq (1). The results are only shown for a number of harmonic functions (*n*) of 1 and 3 to reduce the complexity of the table and show the range of model complexity tested. Annual cycles: 1999/2000-2020/2021.

No. harmonic functions		1	3
**No. observations**		36936	36936
**AIC**		40767	39225
**BIC**		41346	40538
**Effective DF**		67.97	154.09
**Tweedie power**	p	1.59	1.59
**Dispersion**	*ϕ*	10.69	9.16
**Linear parameters**	*β*_*0*_	-2.43	-7.87
	*β*_*c*,*y*_	[-8.56, -2.68]	[-40.44, -2.49]
	*β*_*s*,*y*_	[+0.75, +3.77]	[+0.27, +23.89]
	*β*_*c*,*2*,*y*_	-	[-17.69, +3.39]
	*β*_*s*,*2*,*y*_	-	[-1.21, +15.53]
	*β*_*c*,*3*,*y*_	-	[-3.11, +2.57]
	*β*_*s*,*3*,*y*_	-	[-2.01, +2.53]

**Fig 1:**
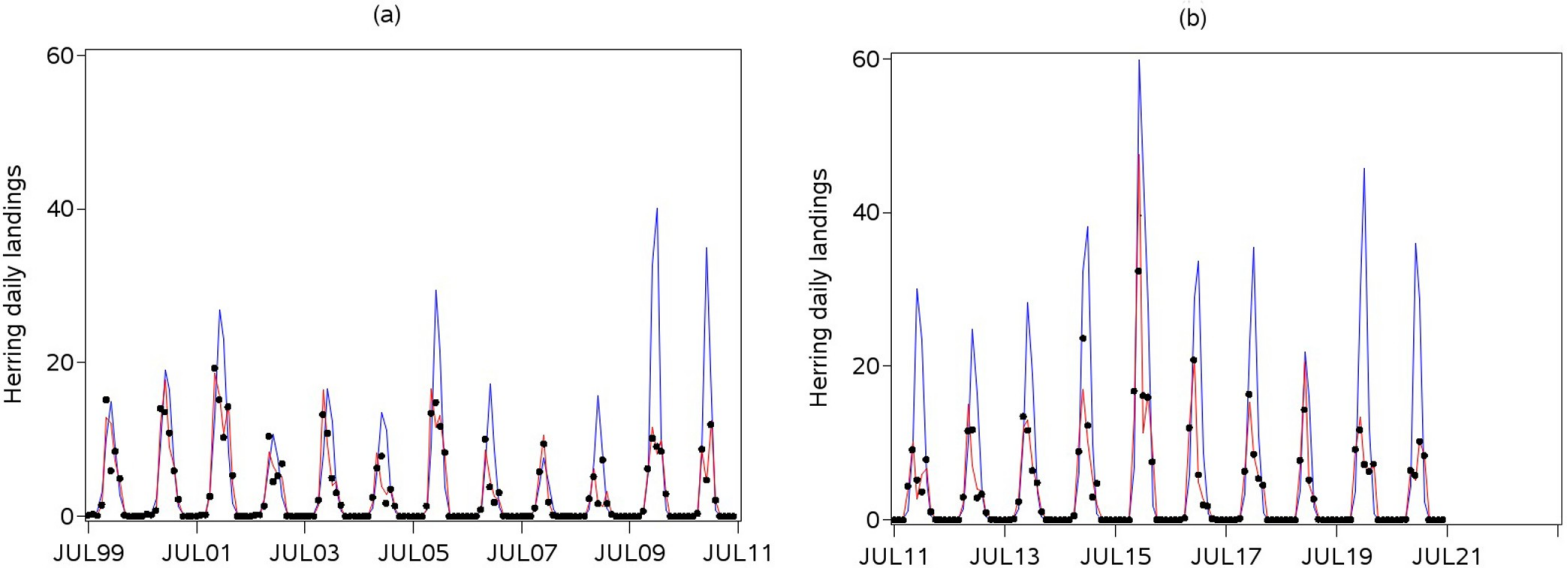
Herring average daily landings (tonnes) per month and per annual cycle: (a) from 1999/2000 to 2010/2011, (b) from 2011/2012 to 2020/2021; observed (black dots), and modelled with GAM. The results are only shown for a number of harmonic functions of one (blue curve) and three (red curve) to reduce the complexity of the figure and show the range of model complexity tested.

The model fit, and the improvements brought about by increasing the number of harmonic functions, was illustrated by comparing observed and predicted average landings (Fig 1). The least parameterized model (*n* = 1) roughly picked up both seasonal and inter-annual patterns during the first annual cycles (from 1999/2000 to 2007/2008), but not afterwards. The fit became particularly poor after 2009/2010, with both an overestimation of the expected peak timing and of the corresponding landings in most annual cycles. In contrast, the model building in three harmonic functions fitted observations well, and that was retained to derive phenological indicators of spawning season.

#### Spawning phenology and duration of Downs herring

**Fig 2:**
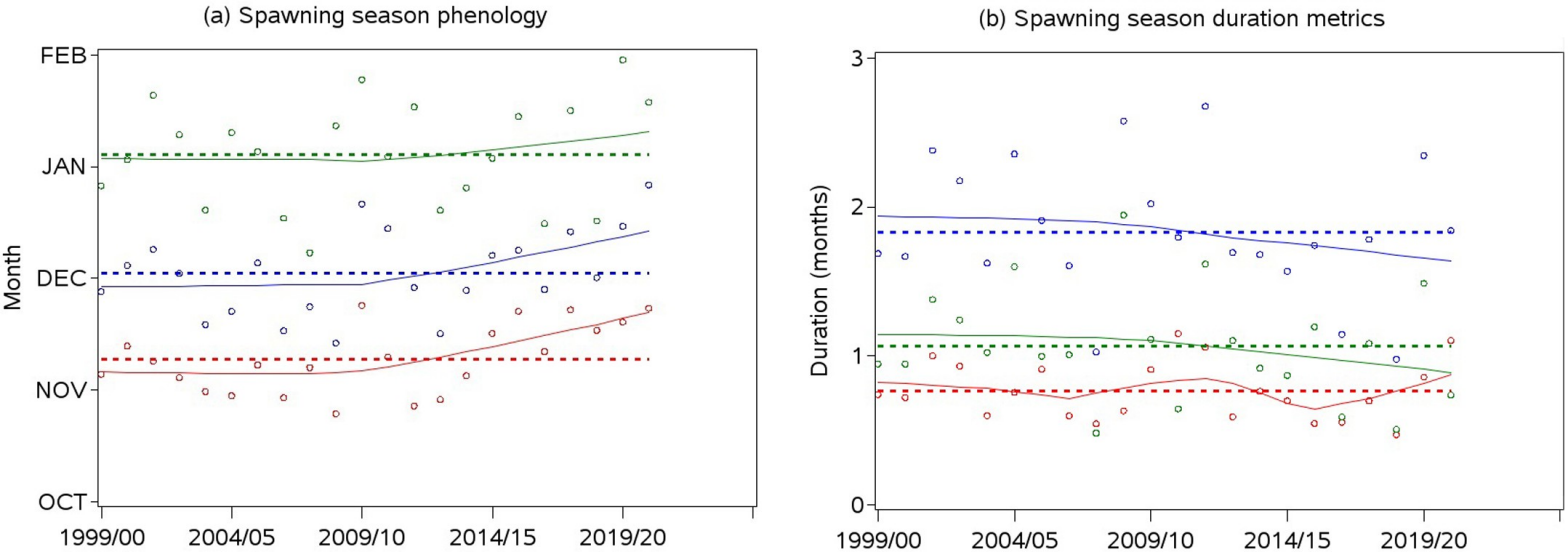
Herring phenological indicators per annual cycle estimated with Eq (1) and three Fourier harmonic functions: (a) phenological indicators reflecting the timing of spawning season: start (red), midpoint (blue), and ending (green); (b) spawning duration metrics (in months): full duration (blue), time elapsed between the start and the midpoint (red), time elapsed between the midpoint and the ending (green); expected values: circles; smoothed values: plain lines; annual averages: dotted lines.

The start and midpoint of Downs herring spawning season fluctuated without trend between 1999/2000 and 2009/2010, and were then delayed until 2020/2021 (Fig 2A). Between 1999/2000 and 2020/2021, the start of spawning season shifted on average from early to mid-November, while the midpoint was delayed from late-November to mid-December. The ending of Downs herring spawning season varied between mid-December and mid-January, and it followed a similar trend to the season’s start and midpoint, although the increasing trend was less pronounced after 2009/2010. The spawning seasonal pattern was asymmetrical, as the time elapsed between the ending and the midpoint was generally larger than the time elapsed between the midpoint and the start of the signal (Fig 2B). The duration of the time spent on spawning grounds was highly variable around a 1.8 month average, and subject to a slightly decreasing trend, which accelerated after 2009/2010 (Fig 2B).

### Annual changes in the spawning phenology and duration of Downs herring in relation to environmental fluctuations

The outputs from the ARMAX models are shown in Table 2.

**Table 2:** ARMAX models explaining the annual variations in Downs herring spawning midpoint, start, ending and duration in relation to autoregressive (AR) and moving average (MA) factors, as well as external variables at different time lags shown in brackets (*SSTAw* = annual sea surface temperature anomaly on wintering spawning grounds, *CALf* = annual changes in the abundance of *Calanus finmarchicus*; *CALh* = annual changes in the abundance of *Calanus helgolandicus*). All the explanatory variables significantly (*p* < 0.05) cross-correlated to the phenological metrics are reported in the “External variables” column, although only those retained in the final model are shown in the “Final ARIMAX model” column.

Period	Phenological index	AR/MA parameters	External variables	Final ARMAX model
1999/00-2020/21	Midpoint (50%)	-	*CALf (0)*, *CALf(1)*	** ${\hat{\theta }}_{y}=4.67+0.04\;CALf_{y}+\eta_{y}$ **
(N = 22)	Start (25%)	-	*CALf (0)*	${\hat{\theta }}_{1,y}=4.01+0.03\;CALf_{y}+\eta_{y}$
	Ending (75%)	-	*CALf(0)*, *CALh(0)*, *SSTAw(1)*	$\begin{array}{l} {\hat{\theta }}_{2,y}=5.94+0.04\;CALf_{y}\\ -0.32\;{\mathrm{SSTAA}}_{y-1}+\eta_{y} \end{array}$
	Duration (25%-75%)	AR(1)	*CALh(0)*, *SSTAw(1)*	* $\begin{array}{l} {\hat{\delta }}_{y}=1.75-0.50\;{\hat{\delta }}_{y-1}+0.03\;CALh_{y}\\ -0.45\;SSTA_{y-1}+\eta_{y} \end{array}$ *
	Start (5%)	-	*CALf(0)*	** ${\hat{\theta }}_{1,y}=3.30+0.02\;CALf_{y}+\eta_{y}$ **
	Ending (95%)	-	*CALf(0)*	* ${\hat{\theta }}_{2,y}=7.03+0.03\;CALf_{y}+\eta_{y}$ *
	Duration (5%-95%)	AR(1)	*SSTAws(1)*	${\hat{\delta }}_{y}=4.33-0.46\;{\hat{\delta }}_{y-1}-0.54\;SSTAw_{y-1}+\eta_{y}$

We first describe the results obtained with the base model, where the percentage of cumulated predicted landings used to estimate spawning start and ending were of 25% and 75%, respectively. The spawning start, midpoint and ending series were not characterized by significant (p < 0.05) auto-correlation, resulting in the absence of AR or MA factors. A first-order autoregressive autocorrelation was found in the spawning duration series. None of the spawning phenology metrics were correlated with April-September SST anomalies on feeding grounds. However, October-December SST anomalies on spawning grounds were negatively correlated with spawning ending and duration experienced one year later. Increased densities of *Calanus finmarchicus* in April-September were concomitant with a delay in the start, midpoint and ending of the spawning season within the same annual cycle. *Calanus finmarchicus* densities were also correlated with spawning midpoint with a lag of one year, but these were not retained in the final ARMAX model. Delays in the ending of spawning season were also correlated with increased densities of *Calanus helgolandicus* within the same annual cycle, when *Calanus finmarchicus* densities were not included as explanatory variable. When the densities of the two *Calanus* species were included only *Calanus finmarchicus* densities had a significant effect on the ending of spawning season. This collinearity effect results from the correlation between the densities of the two *Calanus* species. Densities of *Calanus helgolandicus* taken alone had no statistical effect on the start and the midpoint of spawning season. The best model (judging by AIC values) for spawning season’s start, and midpoint always included *Calanus finmarchicus* densities, with no lag effect, as unique explanatory variable, even when other explanatory variables (e.g., *Calanus helgolandicus* densities) were statistically correlated. The best model for spawning season’s ending included as explanatory variables: *Calanus finmarchicus* densities (with nolag effect) and October-December SST anomalies on spawning grounds in the year before, while *Calanus helgolandicus* densities were statistically correlated (but not retained in the final run). The best model for the duration of spawning season included as explanatory variables: *Calanus helgolandicus* densities (with no lag effect) and October-December SST anomalies on spawning grounds in the year before.

Table 2 also shows the sensitivity of the ARMAX models to changing the percentage of cumulated predicted landings used to estimate spawning start (using 5% instead of 25%) and ending (using 95% instead of 75%). The ARMAX model for spawning start was robust to using the new phenological metrics, which only resulted in moderate changes in the parameter estimates, while the same set of explanatory variables was retained in the final model. The ARMAX model of spawning ending still included *Calanus finmarchicus* densities (with no lag) as a statistically significant explanatory variable, but October-December SST anomalies on spawning grounds in the year before were not retained anymore in the final model. Finally, the ARMAX model of spawning duration still included a first-order autoregressive parameter and October-December SST anomalies on spawning grounds in the year before as statistically significant explanatory variables, but *Calanus helgolandicus* densities (with no lag) were not retained anymore in the final model.

**Fig 3:**
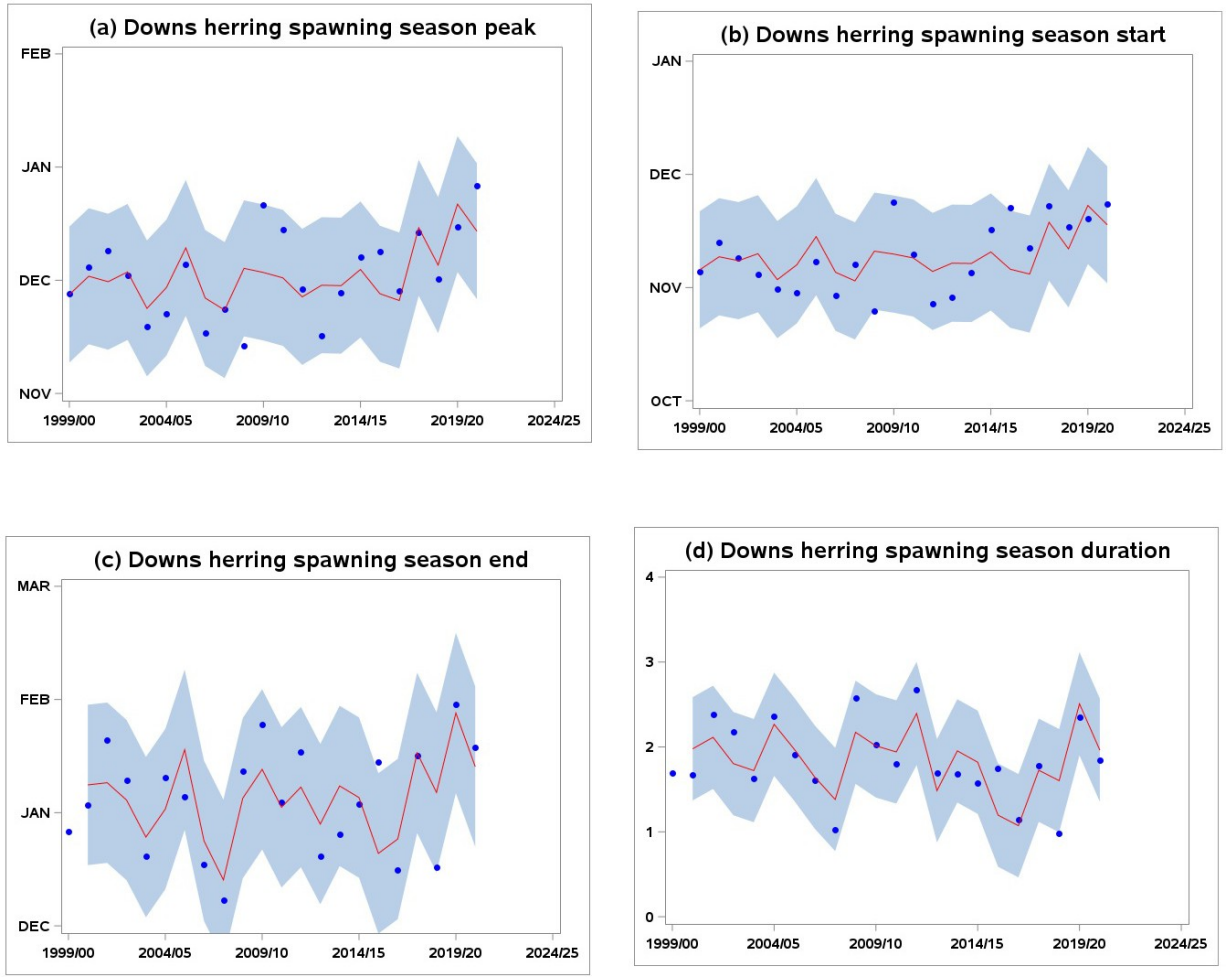
Time series of Downs herring spawning phenological indicators (a) midpoint, (b) start, (c) ending, and (d) duration (decimal months), over the period 1999/00-2020/21. Blue dots: observed values; red line: ARMAX estimates; grey shaded area: inter-quartile [5%, 95%] confidence interval.

The examination of Fig 3A–3D suggested that the ARMAX models picked up some of the phenological variability in Downs herring spawning season. The majority of observations were within the confidence interval of the ARMAX model, suggesting relatively good model performance despite part of the variability remaining unaccounted for. The models were in particular able to reflect the shift occurring in the second half of the time series, after which the overall timing of spawning season has become increasingly delayed.

## Discussion

Our study sheds light on the spawning phenology of winter-spawning Downs herring and its intricate relationship with the environment. In contrast to the many studies demonstrating an earlier and sometimes more extended spawning period for spring-spawning fishes in response to warming waters [8, 11, 16–20], our study reveals that in the case of a winter-spawning population such as Downs herring, a generally contrary trend could be observed. Our study contributes to a novel perspective in the broader understanding of the intricate interplay between herring and its environment, including the influence of predator-prey relationships in shaping herring spawning seasonal patterns.

### Phenological variations in Downs herring spawning in relation to the environment

We first showed that the start, midpoint and ending of Downs herring spawning season have been fluctuating without trend from 1999/2000 to 2009/2010, whereas increasing delays were then observed in the three phenological indicators over the period 2010/2011-2020/2021, a process which was positively correlated with *Calanus finmarchicus* abundance. While a causal effect could not be fully demonstrated, this result was robust to the metrics used to estimate spawning start and ending, and it has to be related to the ecological relevance of *Calanus finmarchicus* as a major feeding source for herring in spring and summer [15, 47, 56]. First, the spatial distribution of Downs herring feeding grounds overlaps that of *Calanus finmarchicus*, which has an affinity for the boreal waters found at high latitudes [52, 53, 57]. Second, selective feeding on nutritious *Calanus finmarchicus* seems to be a common pattern shared with other herring components, e.g., Norwegian spring spawners [58, 59]. One could then hypothesize that high abundance of *Calanus finmarchicus* could incentivize herring pre-spawners to remain longer in more productive feeding grounds, and delay their spawning migrations [14, 60].

The fact that *Calanus finmarchicus* abundance in one annual cycle was cross-correlated with the midpoint of Downs herring spawning season occurring in the following annual cycle should also be commented in the light of some assumptions that have been formulated in the past literature on herring behavior. [14] suggested herring could adapt non-instantaneously (possibly up to several years) to shifts in their prey abundance, preventing herring to over-react to short-time fluctuations in food abundance. Such conservative behavior might explain the 1-year lag we found in the correlation between herring spawning midpoint and *Calanus finmarchicus* abundance. The following year, fish may spawn later in order to optimize reproductive success and/or to avoid a mismatch between spawning timing and optimal environmental conditions for offspring survival, e.g., thermal preferendum of embryos [61, 62].

The abundance of *Calanus helgolandicus* had no statistical effect on the start and the midpoint of spawning season, but it was positively correlated with delays in the ending of spawning season, and also retained in the final spawning duration ARMAX model. The extent to which *Calanus helgolandicus* abundance may affect the spawning phenology of Downs herring should, however, be interpreted cautiously for two reasons. First, we showed that the abundance time series of *Calanus finmarchicus* and *Calanus helgolandicus* were correlated over the period 1999–2020. Second, the abundance of *Calanus finmarchicus* could be underestimated in summer, a period where this copepod moves deeper to access colder waters [63], where it would be poorly sampled but still represent an accessible and nutritious prey to herring schools. In other words, the effect of *Calanus finmarchicus* on the behaviour of Downs herring spawners might partially hide behind that of *Calanus helgolandicus* over the period 1999–2020.

Still, it is striking that *Calanus helgolandicus* densities were correlated with the ending, and not the start or the midpoint, of spawning season. Despite the caveats expressed above, there are also ecological reasons to support that *Calanus helgolandicus* could play a role in the phenology of herring spawning migrations, especially at the end of the process. One such reason could be that the diet of Downs herring varies and possibly diversifies during the time interval when early and late spawners migrate from the northern North Sea feeding areas to the EEC-SNS spawning grounds. The apparent peak of abundance of *Calanus finmarchicus*, i.e., in May, occurs before that of *Calanus helgolandicus*, i.e., in September (S1 Text). When the abundance of *Calanus finmarchicus* is decreasing, herring could be forced to search opportunistically for alternative prey available, such that the phenology of late spawners be more dependent on prey (e.g., *Calanus helgolandicus*) emerging after *Calanus finmarchicus*, compared to early spawners. Although *Calanus helgolandicus* has a more southerly distribution than *Calanus finmarchicus*, increased sea temperatures could have forced this copepod to gradually migrate northwards over the years [64], where it would be increasingly accessible to herring schools at the time they complete their spawning migrations. The underlying mechanism could also be more complex, as diet shifts resulting from changes in prey availability and lipid composition could affect, in addition to direct temperature effects, the gonadal maturation process and eventually the timing of spawning migrations [10]. This diet-switching hypothesis could be investigated by sampling herring stomach contents and maturity stages sequentially, along their feeding-to-spawning migration corridor. When spawning ending was estimated at a later period (based on the 95% of cumulated predicted herring landings), the abundance of *Calanus finmarchicus* had no significant effect on either spawning ending or duration, possibly due to the low abundance of the copepod at that time.

Although *Temora longicornis* was about ten times more abundant than both *Calanus finmarchicus* and *Calanus helgolandicus* in the feeding area, this copepod did not appear to constrain the timing or the duration of Downs herring spawning season. This may be attributed to the comparatively lower size and lipid content of *Temora longicornis* [65]. Notably, given the crucial role of lipid-rich prey for the energetic requirements of Downs herring, especially as a capital breeder, the higher lipid content in *Calanus finmarchicus*, rather than sheer abundance, could emerge as a pivotal factor influencing the interactions between copepods and the reproductive timing of herring [66].

It was unexpected that April to September sea temperature experienced by herring on its feeding grounds, as implemented in the ARMAX models, had no statistical effect on any spawning phenology metric. It was also not clear why increased sea temperatures experienced by herring on the Downs spawning grounds in October-December in one year were correlated with an earlier spawning ending and a shorter spawning duration in the following year. Reproduction is one of the most temperature-sensitive processes and temperature requirements for adult spawners have been recognized as a critical bottleneck in the life cycle of fish [7], and one would expect this to affect both the timing and duration of spawning season. Temperature more particularly affects age at first maturation and gonadal development. Gonad maturation could be accelerated by increased temperatures, in herring and other fish species [9, 10, 67]. In contrast, temperatures increasing above optimal maturation window could adversely affect endocrine processes and delay gametogenesis [26, 68–70], and possibly induce a behavioral response [27]. The results of these investigations show that sea warming could affect the timing of spawning season, in a direction depending on whether and how temperatures lie within or beyond a population/stock component’s preferendum. Finally, temperature could have affected herring spawning phenology indirectly through a variety of processes, including the phenology of zooplankton [3, 30]. This could suggest that the effects of sea temperature on the spawning phenology of Downs herring were complex and could perhaps not be implemented as a linear effect, as assumed in the ARMAX models.

It is noteworthy that our conclusions on the linkages between spawning phenology metrics and environmental factors were drawn from a 22-year period (1999–2021), with fluctuating and positive sea surface temperature anomalies, increased abundance of *Calanus finmarchicus*, and high abundance of *Calanus helgolandicus*. It remains to be seen the extent to which these conclusions would hold with different environmental trends, such as those observed in the earlier period (e.g., 1980–1999): increased temperature, decreasing abundance of *Calanus finmarchicus*, low abundance of *Calanus helgolandicus*.

The asymmetry of the landing time series suggests that two separate processes are associated to the start and ending of spawning season. This is reflected by the number of Fourier harmonic functions retained in Eq (1). Indeed, a model including only one harmonic function, implying a symmetrical increase and decrease around peak timing for each annual cycle, did not match our landings data well, while increasing the number of harmonic functions to three, and thereby allowing for an asymmetrically-shaped seasonal signal, resulted in a substantially improved fit. While the processes underlying the differential paces characterizing the start and ending of spawning season should be further investigated, the diet-switching assumption we discussed above could provide a possible explanation. Another assumption would be that other covariates should be considered to further explain the spawning phenology of Downs herring, including alternative environmental parameters [5, 71], as well as the structure and dynamics of the spawning population itself.

### Phenological variations in Downs herring spawning season in relation to population factors

The spawning phenology of species such as herring may not only be affected by environmental factors, but also by fish ontogeny [8, 11, 67, 72, 73]. These studies suggested that age-at-maturity, gonad development as well as spawning could be stock-, size- and/or age-dependent, and could vary from one herring individual to another, with likely consequences on both spawning timing and duration. [8, 11, 72] showed that larger and older fish would spawn earlier and possibly over a longer time period compared to smaller and younger individuals. Whether this finding would apply to winter-spawning Downs herring remains to be investigated. Still, the fact that some of the fitted annual cycles were bimodal (Fig 1) is an indication that several cohorts could migrate at different periods. While neither size/age herring distributions could be made available over the whole period being investigated, their inclusion could be considered in future studies based on shorter time periods. The conclusions of our study could be even strengthened by inputting data time series of sexual maturity specific to the Downs herring component. While Downs herring sexual maturity information has been only irregularly collected in its most southerly distribution area, close estimates may possibly be retrieved by relating herring gonad development to known covariates for which time series are available. One such covariate could be herring fat contents, on which information has routinely been collected by some herring processing industries over long time periods [74].

### Back to the methods

A novel two-tiered method was applied to evaluate inter-annual phenological variations in Downs herring spawning season, and then to relate these to environmental drivers. GAMs building in several Fourier harmonic functions and a non-linear annual effect were applied to model variations in herring landings. The harmonic equations, consisting of sine waves with year-dependent coefficients aimed at filtering out the predominant seasonal signal, such to reveal inter-annual phenological changes. Our approach is of intermediate complexity between purely statistical modelling and a full conceptual representation of all processes and ecological compartments relating temperature variations to herring abundance and phenology dynamics. With a fully conceptual model, seasonal temperature variations would propagate into the different ecosystem compartments (including zooplanktonic prey) through a set of differential equations [36, 75, 76], instead of being forced directly into herring landings.

Our study builds on an analysis of herring landings, which was the only source of information available reflecting both inter- and intra-annual abundance patterns in herring (e.g., research surveys focused on North Sea herring are not designed to capture seasonal or monthly patterns). Inter-annual landing variations comprise an element of fish biomass and an element of fishing pressure, and the series are temporally auto-correlated. These are therefore difficult to interpret and have been integrated in the model as a non-linear effect. We then extracted the year-dependent seasonal patterns of herring landings using harmonic functions, from which we derived inter-annual variations in the spawning phenology of Downs herring. This approach built on two main assumptions: (1) fishers had a perfect knowledge of where and when to fish and, (2) they could fish with limited external constraints.

Over the period being investigated, fishers have been equipped with and made extensive use of electronic acoustic devices, so assuming they had the technical ability to locate herring schools adequately is reasonable [77, 78]. Still, some fishers have varied skills, vessels and onboard equipment, and some may be better able to locate and harvest fish than others [42, 79]. While we reduced heterogeneity across fishing vessels and fishers as much as possible by restricting our dataset to the pelagic trawlers operating short fishing trips, we did not remove it completely, an aspect which could be further investigated as an extension to the present study. We also reduced the geographical range of our study, so the distance to fishing grounds may not be considered as a major limiting factor [80]. Reducing the geographical range further would have been possible but at the expense of less fishing trips available throughout the period being investigated, which would have compromised the estimation of the numerous parameters required in our analyses.

Other external constraints that may potentially apply to the herring fishery are biomass availability and management regulations. North Sea herring has been exploited sustainably over the period 1999–2021 [41], so biomass availability was not considered constraining. However, annual catch quotas could restrict herring fishing when approached at the end of the year. French quota consumption could be made available over a shorter time period, 2012–2019 ([81], Fig S9 in Supplementary Material). This suggested that French herring quota uptake was high but not exceeded, and also that it fluctuated without trend over that period. Also, the effects of quota constraints may have been alleviated by quota-swapping among countries and buybacks, which would be facilitated in a period when North Sea herring is harvested sustainably.

### Consequences of a phenological shift in spawning season

Phenological shifts in Downs herring spawning could adversely affect the survival of newborn herring eggs and larvae, and delving into recruitment dynamics. Downs herring born later in the spawning season could experience an unfavorable abiotic and biotic environment, starve and die before contributing to recruitment [2, 82]. Temporal shifts in spawning time would cascade down to early life stages of fish who would be confronted to day length and temperatures different from the normal, with consequent effect on their growth rates and survival [83, 84]. In addition, shifts in the spawning season were also shown to impact quality of gametes in spawners [69], which could impair fertility, fertilization and/or be detrimental to embryo survival and development [68, 85].

Phenological changes in herring spawning would also have bottom-up effects and affect predation by other fish as well as fishing activities. Such processes could be investigated by using end-to-end ecosystem models developed in the study area [86, 87], and possibly building in fishers’ behavior mechanisms [88].

## Supporting information

S1 Fig Map of the North Sea and the English Channel. Downs herring is assumed to feed in spring and summer in the grey shaded area, corresponding to CPR Standard Areas B2+C2, before spawning in autumn-winter in the Eastern English Channel and the Southern North Sea (EEC-SNS). The blue area shows the areas visited by French pelagic trawlers operating less than two days in EEC-SNS.
(DOCX)

S1 Text Exploration of the trends, seasonal patterns and correlation within and across biotic and abiotic environmental variables. (DOCX)

S2 Text Modelling annual and monthly variations in the total length of pelagic trawlers operating in the Eastern English Channel and the Southern North Sea. (DOCX)
