# Supplementary material for: Effects of zooplankton abundance on the spawning phenology of winter-spawning Downs herring (Clupea harengus)
Source: PLoS One. 2025 Feb 5;20(2):e0310388. doi: 10.1371/journal.pone.0310388 (PMC11798473; doi:10.1371/journal.pone.0310388)
Supplement: S1 Fig — Downs herring is assumed to feed in spring and summer in the grey shaded area, corresponding to CPR Standard Areas B2+C2, before spawning in autumn-winter in the Eastern English Channel and the Southern North Sea (EEC-SNS). The blue area shows the areas visited by French pelagic trawlers operating less than two days in EEC-SNS. (DOCX) [file pone.0310388.s001.docx]

**S1 Fig.** Map of the North Sea and the Eastern English Channel. Downs herring is assumed to feed in spring and summer in the grey shaded area, corresponding to CPR Standard Areas B2+C2, before spawning in autumn-winter in the Eastern English Channel and the Southern North Sea (EEC-SNS). The blue area shows the areas visited by French pelagic trawlers operating less than two days in EEC-SNS.

**
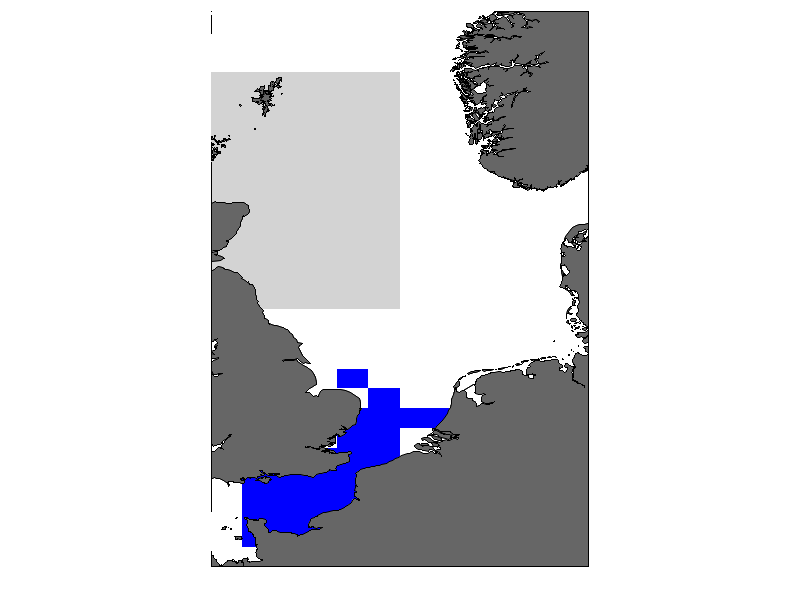
**
