# Supplementary material for: Effects of zooplankton abundance on the spawning phenology of winter-spawning Downs herring (Clupea harengus)
Source: PLoS One. 2025 Feb 5;20(2):e0310388. doi: 10.1371/journal.pone.0310388 (PMC11798473; doi:10.1371/journal.pone.0310388)
Supplement: S1 Text — (DOCX) [file pone.0310388.s002.docx]

**S2 Text. Exploration of the trends, seasonal patterns and correlation within and across biotic and abiotic environmental variables.**

Fig A.a shows a steep increase of SST anomalies over the period 1980-2021, with positive annual SST anomalies since 1997 in all years except 2010 (October-December spawning grounds) and 2012 (April-September feeding grounds). Despite a consistent positive trend over the whole period, the two SST anomalies series were subject to different inter-annual dynamics. Subject to strong inter-annual variations, the smoothed abundance of *Calanus finmarchicus* decreased over the period 1980-2000, increased slightly (2000-2013), and then steeply (2013-2020) (Fig A.b). The smoothed abundance of *Calanus helgolandicus* increased slightly (1980-1997), then steeply (1997-2009), decreased (2009-2015), and increased steeply again (2015-2020) (Fig A.c). Finally, the smoothed abundance of *Temora longicornis* increased steeply (1980-1983), decreased steadily (1983-2007), increased (2007-2011) and decreased (2011-2020) (Fig A.d).

**Fig A.** Annual time series of environmental parameters: (a) 1980-2021 SST anomalies averaged over the main North Sea herring feeding grounds in April-September (blue line), and over an area covering Downs spawning grounds in October-December (red line); (b, c, d) 1980-2020 CPR zooplankton counts (average numbers per 3 m^3^ filtered seawater): (b) *Calanus finmarchicus*, (c) *Calanus helgolandicus*, (d) *Temora longicornis.* Circles: input data; plain lines: smoothed values; dotted lines: baseline (SST anomalies) or annual average (zooplankton counts).

| 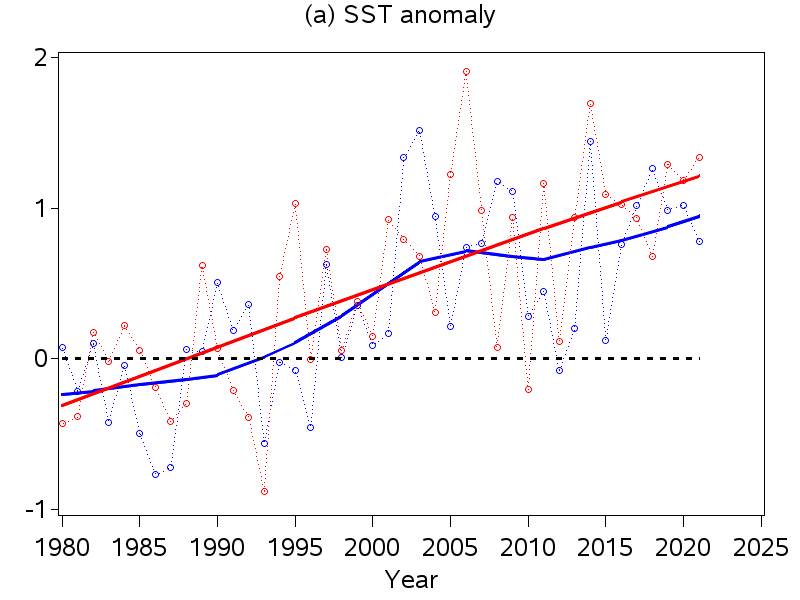 | 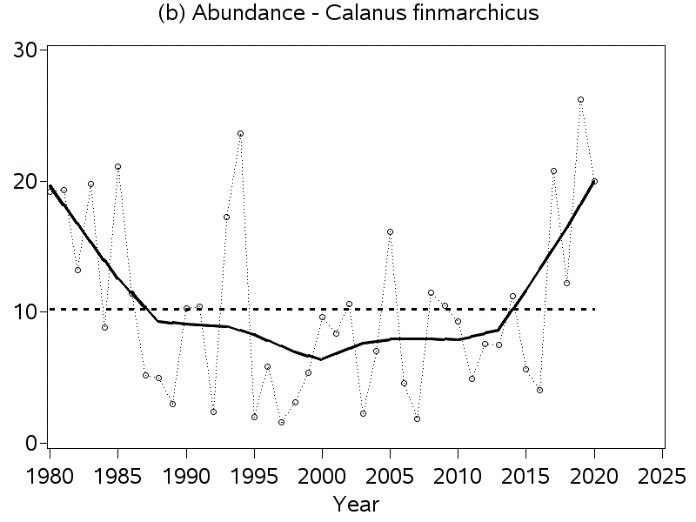 |
| --- | --- |
| 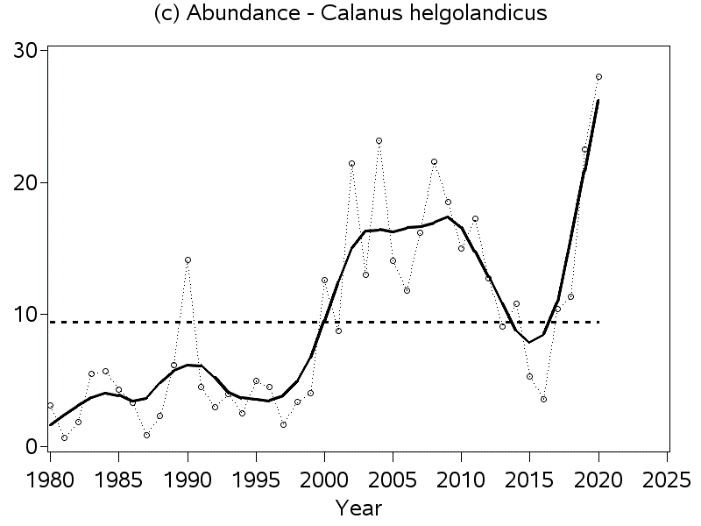 | 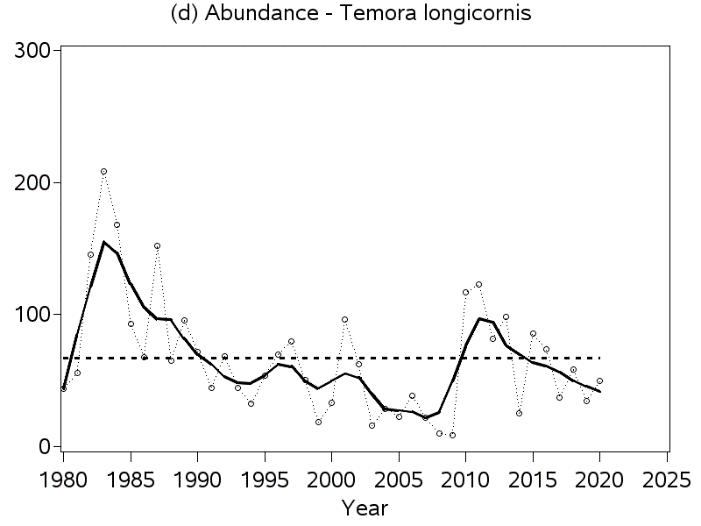 |

Fig B.a shows that *Calanus finmarchicus* abundance sampled by the Continuous Plankton Recorder in Standard Areas B2+C2 is essentially distributed in spring and summer, with a peak in May. The abundances of *Calanus helgolandicus* (Fig B.b) and *Temora longicornis* (Figure B.c) peak later in the year, in September. Note that while the true peak for *Calanus helgolandicus* is in September, the seasonal pattern is bimodal with an inferior mode in May matching that of *Calanus finmarchicus*.

**Fig B.** Monthly CPR zooplankton counts (average numbers per 3 m^3^ filtered seawater) averaged over the period 1999-2020: (a) *Calanus finmarchicus*, (b) *Calanus helgolandicus*, (c) *Temora longicornis*.

| **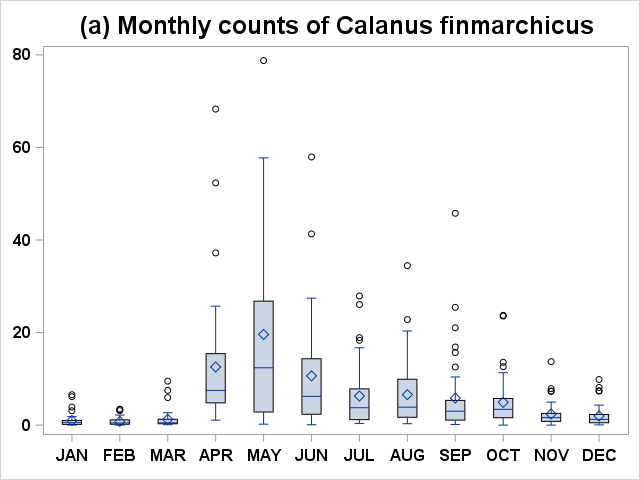** | **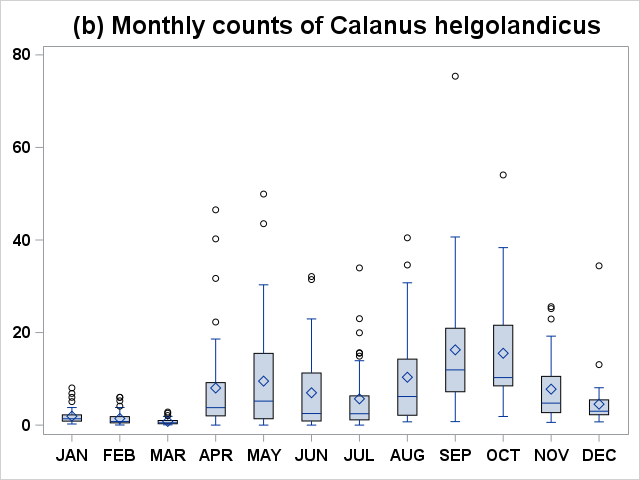** |
| --- | --- |
| **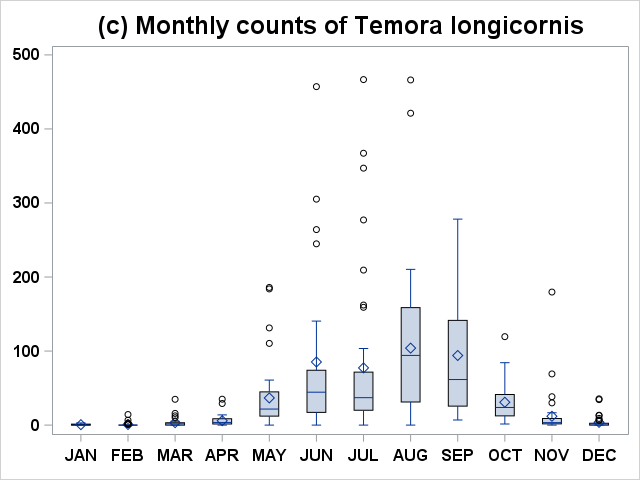** |  |

We examined the temporal structure inherent in the different environmental variables (SST anomalies, zooplankton abundances), and the common patterns they may share, which could blur the interpretation of their possible effect in driving the phenology of spawning migrations. A preliminary analysis was carried out by checking temporal autocorrelation within and cross-correlation between the environmental time series over the period 1999-2020. As shown in the Table below, none of the environmental factors being investigated were subject to significant autocorrelation at any time lag. Cross-correlation were found at lag 0, and not for any other lag, between the abundances of *Calanus finmarchicus* and *Calanus helgolandicus* (positive cross-correlation), and also between the abundance of *Temora longicornis* and SST anomalies in April-September over herring feeding grounds (negative cross-correlation). The collinearity across the abundances of both *Calanus* species may result from the spring alignment between the peak of *Calanus finmarchicus* and the inferior mode of *Calanus helgolandicus* abundances. None of the other pairs of environmental variables were subject to significant cross-correlation at any time lag.

**Table.** Autocorrelation within and cross-correlation between the different environmental factors at different time lags (SSTAs = SST anomaly in April-September over the main feeding grounds; SSTAw = SST anomaly in October-December over Downs spaning grounds; CALf = abundance of *Calanus finmarchicus*; CALh = abundance of *Calanus helgolandicus*; TEMl = abundance of *Temora longicornis*). Only the sign of significant (p < 0.05) auto- or cross-correlation is displayed (with the lag in brackets); ns=”non-significant”. Autocorrelation and cross-correlation are qualitatively displayed in the diagonal and in the bottom-left triangle of the table, respectively.

|  | SSTAs | SSTAw | CALf | CALh | TEMl |
| --- | --- | --- | --- | --- | --- |
| SSTAs | ns |  |  |  |  |
| SSTAw | ns | ns |  |  |  |
| CALf | ns | ns | ns |  |  |
| CALh | ns | ns | +(0) | ns |  |
| TEMl | -(0) | ns | ns | ns | ns |
