# Supplementary material for: Effects of zooplankton abundance on the spawning phenology of winter-spawning Downs herring (Clupea harengus)
Source: PLoS One. 2025 Feb 5;20(2):e0310388. doi: 10.1371/journal.pone.0310388 (PMC11798473; doi:10.1371/journal.pone.0310388)
Supplement: S2 Text — (DOCX) [file pone.0310388.s003.docx]

**S3 Text. Modelling annual and monthly variations in the total length of pelagic trawlers operating in the Eastern English Channel and the Southern North Sea.**

Vessel length of pelagic trawlers was modelled using Generalized Additive Models building in a tensor product to accommodate the combination of annual and monthly effects, assuming a Gamma distribution. For each observed fishing vessel *v*, the expected value of vessel length *E[Length_v_]* could be formulated by the Equation below:

$E\left[ {Length}_{v} \right]=exp\left\{ a+sp(y,t) \right\}$)

Where *a* is a constant base line, y is an annual cycle; t is the month within each annual cycle. The predicted vessel length resulting from the GAM is shown below.

**Fig.** Contours of predicted vessel length as a function of annual cycle and month.

**
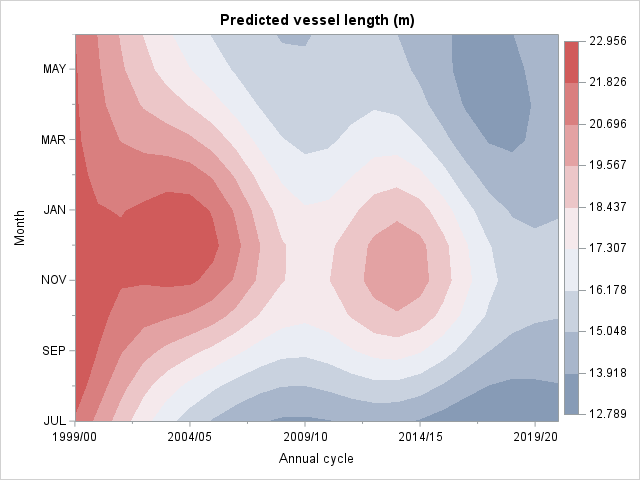
**
